# PW01-020 – MEFV mutations carrier rate in Central Europe

**DOI:** 10.1186/1546-0096-11-S1-A73

**Published:** 2013-11-08

**Authors:** M Debeljak, N Toplak, N Abazi, M Kolnik, B Szabados, V Mulaosmanovic, J Radović, J Vojnović, T Constantin, D Kuzmanovska, T Avčin

**Affiliations:** 1 Center for Medical Genetics, University Children's Hospital, University Medical Center, Ljubljana, Slovenia; 2 Department of Allergology, Rheumatology and Clinical Immunology, University Children's Hospital, University Medical Center, Ljubljana, Slovenia; 3 University Children Hospital, Medical Faculty, Ss. Cyril and Methodius University, Skopje, Macedonia, the Former Yugoslav Republic of; 4 Department of Allergology, Rheumatology and Clinical Immunology, University Children's Hospital, University Medical Center, Ljubljana, Slovenia; 5 Unit of Paediatric Rheumatology, 2nd Department of Pediatrics, Semmelweis University Budapest, Budapest, Hungary; 6 Children’s Hospital University, Clinical Center , Sarajevo, Bosnia and Herzegovina; 7 Department of Pediatric Rheumatology, Faculty of Medicine, University Niš, Niš, Serbia

## Introduction

Familial Mediterranean fever (FMF) is an autosomal-recessive disorder characterized by recurrent attacks of fever and serositis common in eastern Mediterranean population. Over 160 mutations have been identified in MEFV gene responsible for FMF. The most common mutations in MEFV gene are E148Q, M694I, M694V, V726A and M680I. The distribution pattern of MEFV mutation along the Mediterranean Sea is not uniform; eastern populations have the highest number of carriers (20-39%), whereas western Mediterranean populations are practically unaffected.

## Objectives

The aim of this study is to determine the carrier rate in healthy Macedonian, Serbian, Slovene, Bosnian and Hungarian population.

## Methods

We screened 100 healthy subjects from all 5 populations. Exon 10 was PCR amplified and screening was performed with dHPLC. All amplicons with detected nucleotide changes were subsequently sequenced with ABI prism 310 genetic analyzer. Amplicons of exon 2 were directly sequenced.

## Results

Heterozygous mutations were found in 4% of apparently healthy Hungarians, 7% of Slovenians, 8% of Bosnians, 11% of Serbians and in 16% of apparently healthy Macedonians. Mutations found in Hungarian population were as follows: V726A (1), K695R (3). Mutations found in Slovenian population were: V726A (1), K695R (5) and E148Q (1). Mutations found in Bosnian population were: V726A (1), K695R (6) and F756C (1). Mutations found in Serbian population were: E148Q (6), K695R (5). Mutations found in Macedonian population were as follows: E148Q (8), K695R (7) and M694V (1).

## Conclusion

We found higher than expected carrier rate in all populations, from 4% to 16%. It is interesting to note that more than half (60%) of detected carriers in all analyzed populations has K695R mutation.

## Disclosure of interest

None declared

